# Human airway organoids as 3D *in vitro* models for a toxicity assessment of emerging inhaled pollutants: Tire wear particles

**DOI:** 10.3389/fbioe.2022.1105710

**Published:** 2023-01-06

**Authors:** Yingying Jiang, Lin Lu, Chao Du, Yanting Li, Wenting Cheng, Huanhuan Bi, Guo Li, Min Zhuang, Dunqiang Ren, Hongmei Wang, Xiaoya Ji

**Affiliations:** ^1^ Department of Occupational and Environmental Health, School of Public Health, Qingdao University, Qingdao, China; ^2^ Department of Pulmonary and Critical Care Medicine, Affiliated Hospital of Medical College of Qingdao University, Qingdao, China

**Keywords:** human airway organoids, tire wear particles, cell apoptosis, ROS generation, cell composition

## Abstract

Three-dimensional (3D) structured organoids have become increasingly promising and effective *in vitro* models, and there is an urgent need for reliable models to assess health effects of inhaled pollutants on the human airway. In our study, we conducted a toxicity assessment of human airway organoids (hAOs) for tire wear particles (TWPs) as an emerging inhaled pollutant. We induced primary human bronchial epithelial cells (HBECs) to generated human airway organoids, which recapitulated the key features of human airway epithelial cells including basal cells, ciliated cells, goblet cells, and club cells. TWPs generated from the wearing of tire treads were considered a major source of emerging inhaled road traffic-derived non-exhaust particles, but their health effect on the lungs is poorly understood. We used human airway organoids to assess the toxicology of tire wear particles on the human airway. In an exposure study, the inhibitory effect of TWPs on the growth of human airway organoids was observed. TWPs induced significant cell apoptosis and oxidative stress in a dose-dependent manner. From the qPCR analysis, TWPs significantly up-regulated the expression pf genes involved in the inflammation response. Additionally, the exposure of TWPs reduced *SCGB1A1* gene expression associated with the function of the club cell and *KRT5* gene expression related to the function of basal cells. In conclusion, this was first study using human airway organoids for a toxicological assessment of TWPs, and our findings revealed that human airway organoids provide an evaluation model of inhaled pollutants potentially affecting the lungs.

## 1 Introduction

It is estimated that air pollution is responsible for more than two million deaths worldwide each year through damage to the respiratory system (Kim et al., 2015) and 14% of childhood asthma cases; additionally, 15% of all aggravations of childhood asthma in ten European cities are ascribed to exposure to pollutants associated with road traffic (Guarnieri and Balmes 2014). The upper airway epithelium acts as a protective barrier of the respiratory system from the external environment and is continuously exposed to various pollutants such as particulate matter (PM), gaseous pollutants, and mixed traffic-related air pollution. An occupational cohort exposed to diesel engine exhaust (DEE) revealed that workers have a significantly thicker airway wall indicative of airway remodeling and lower spirometry measurements indicative of airway obstruction (H Liu H. et al., 2021). Meng et al. (2022) reported that tri-n-butyl phosphate (TnBP), as a typical flame retardant ubiquitously detected in indoor and outdoor environments, induces airway hyperresponsiveness and the secretion of inflammatory mediators that exacerbate asthma. Thus, adverse effects on the airway induced by air pollutants can lead to a variety of lung diseases, and identifying potential hazard factors that are harmful to the airway and evaluating their toxic effects are of critical significance for the prevention of associated pulmonary disease.

Currently, most *in vitro* approaches to evaluate pollutants’ toxicity on the human airway utilize 2D monolayers. Human cancer cell lines, such as A549, and immortalized cell lines, like BEAS-2B, are widely employed in toxicology studies. For instance, mucus overproduction and an inflammatory response are observed in A549 and BEAS-2B cells after treatment with air pollutants (Albano et al., 2020; Liu et al., 2020). However, cancer and immortalized cells removed from their native microsystems and placed into 2D cultures lead to a loss of tissue-specific function of cells. Conversely, low passage primary human bronchial epithelial cells (HBECs) at an air–liquid interface (ALI) are developed to form a mucociliary epithelium of airways (Haswell et al., 2010), but the limitation of their scarce availability and short-time of expansion has challenged the field.

Recently, the rise of 3D organoids that mimic corresponding *in vivo* organs in drug discovery implicates that human stem cell-derived cells generate an abundant and accessible source of *in vitro* models for chemical toxicity and a safety assessment. Actually, 3D organoids of human airways derived from human induced pluripotent stem cells are technically challenging and time-consuming. Indefinitely propagating 3D human airway organoids (hAOs), which differentiate from primary HBECs, overcomes hurdles that currently and closely resemble key features of airways and that progressively lose proliferative and differentiation potential (Sachs et al., 2019). Thus, hAOs can recapitulate *in vivo* action in a much more simplified and efficient way and offer new perspectives for environmental toxicology.

Tire wear particles (TWPs), which are generated by the wearing of tire treads on roads, are emerging particulate matter that is derived from traffic sources. It is estimated that a total of 5.9 million tons of TWPs are emitted globally each year (Kole et al., 2017). Many studies on airborne TWPs detection provide evidence that TWPs suspend together with road dust and commonly exist in indoor/outdoor dust. For example, average concentrations of 22,581 and 9,818 μg/g of TWPs were detected in industrial and residential area road dust, respectively (Youn et al., 2021). Moreover, TWPs can occur at sizes <10 µm (Harrison et al., 2012), and hence, inhalation exposure might trigger adverse effects on the respiratory system. A recent study revealed that inhalation of TWPs induce restricted ventilatory dysfunction and fibrotic pathological changes in mice (Y Li M. et al., 2022). However, there are few studies on how TWPs cause lung injury and whether they have toxic effects on airways. In this study, we induce primary HBECs to differentiate into hAOs to study the toxicity of TWPs on human airways and evaluate the benefits of hAOs for toxicity studies.

## 2 Materials and methods

**FIGURE 1:**
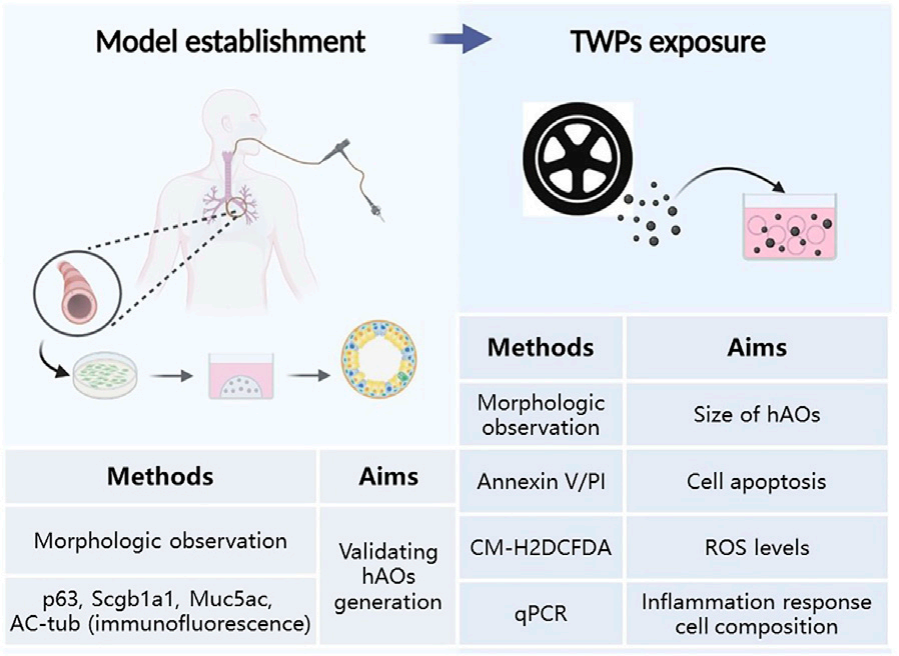
Schematic representation and methods summary of our study.

We conducted model establishment and TWP exposure studies. The experiments and their aims are summarized in Figure 1.

### 2 1 hAO differentiation and culture

Primary human bronchial epithelial cells (HBECs) were obtained from the Department of Pulmonary and Critical Care Medicine, Affiliated Hospital of Medical College of Qingdao University. The use of HBECs was approved by the Ethics Committee of Medical College of Qingdao University (QDU-HEC-2022022). HBECs were maintained at 37°C in 5% CO_2_ and in BEGM (CC-3170, Lonza, United States) with 0.5x fungizone (Gibco, United States). The HBEC pellet was mixed with Corning^®^ Matrigel^®^ Growth Factor Reduced (GFR) Basement Membrane Matrix (Corning, United States), and 50 μl of the Matrigel-HBECs (5 × 10^3^) suspension was allowed to seed on prewarmed 24 well plates for 30 min. After complete Matrigel solidification, PneumaCult™ Airway Organoid Seeding Medium (STEMCELL Technologies, CA) containing 1× penicillin/streptomycin was added for the first 3–7 days. For establishment of the hAOs, the differentiation medium was supplemented for 7 days. Additionally, the culture medium was changed every 3–4 days.

### 2.2 hAO characterization

When models showed the cavity-like structure after 10–14 days of standard culture, the specific protein markers, including Trp63 (p63) for basal cells, acetylated α-tubulin (Ac-tub) for ciliated cells, mucin 5AC (MUC5AC) for goblet cells, and secretoglobin family 1A member 1 (*SCGB1A1*) for club cells, were used to stain different types of cells for characterizing the hAOs. In detail, the basement membrane matrix was depolymerized to harvest organoids from culture, and organoids were minimally washed with PBS and fixed in 4% paraformaldehyde for 10 min, blocked for 30 min, and incubated overnight with the primary antibodies p63 (1:200, ab124762, Abcam, United States), Ac-tub (1:200, T7451, Sigma, United States), MUC5AC (1:200, ab3649, Abcam, United States), and *SCGB1A1* (1:200, NBP2-75705, Bio-Techne, United States). Then, hAOs were minimally washed and incubated with FITC-conjugated secondary antibody (1:200, Zenbio, China) or Cy3-conjugated secondary antibody (1:200, Zenbio, China) for 2–3 h. Finally, organoids were washed and incubated with DAPI stain for 10 min in the dark and were imaged by a confocal microscope (Leica, TCS SP8, Germany).

### 2.3 TWP preparation and characterization

The TWPs preparation method was referred to in our previous studies (Y Li M. et al., 2022). In short, a tire had dust removed by grinding with a carbon steel flat file, and the particles were placed into a centrifugal tube containing liquid nitrogen and ground with steel balls for 5 min. A total of 50 ml deionized water was added to the fine TWPs, following filtration with a 40-μm filter after ultrasonic dispersion. Finally, the TWP suspension was transferred to a freeze-drying bottle and then frozen, vacuumed, and the TWPs were obtained. A Sirion 200 scanning electron microscope (SEM, FEI, Holland) was used to evaluate the size and morphology of TWPs.

### 2.4 Cell viability

HBECs were placed in a 96-well plate with BEGM (CC-3170, LONZA, United States) at a density of 1 × 10^4^ cells per well. After the HBECs were allowed to attach for 24 h, the test medium (BEGM containing the test TWPs concentrations) was replaced and exposure permitted for 48 h. Then, HBECs were minimally washed with PBS and stained with DAPI. Images were captured using a confocal microscope (Leica, TCS SP8, Germany). The collected images were analyzed by the Image-Pro Plus 6.0 software to determine the number of cells. All tests were performed with three replicates.

### 2.5 The hAO exposure to TWPs

Models showed the cavity-like structure after 10–14 days of standard culture. The basement membrane matrix was depolymerized to harvest organoids, and organoids were slightly washed with PBS and cultured with the test medium (PneumaCult™ Airway Organoid Medium containing the test TWPs concentrations) for 48 h. Finally, the morphology of hAOs was captured under bright field using a confocal microscope (Leica, TCS SP8, Germany). All tests were performed in three replicates. The collected images were analyzed by Image-Pro Plus 6.0 software to determine the size of hAOs.

### 2.6 ROS production measurement

After depolymerized hAOs were cultured with the test medium for 48 h, ROS levels were measured by 6-chloromethyl-2′,7′-dichlorodihydrofluorescein diacetate, acetyl ester (CM-H2DCFDA, Invitrogen, United States), and then the hAOs were washed off with PBS and incubated for 30 min with 5 μM CM-H2DCFDA in 37 °C. BSA was added to block for 10 min, and the hAOs were incubated with DAPI stain for 10 min in the dark. Images were obtained using a confocal microscope (Leica, TCS SP8, Germany). All tests were performed in three replicates, and the collected images were analyzed by the Image-Pro Plus 6.0 software to quantify the ROS levels in hAOs.

### 2.7 Annexin V/PI assay

After depolymerized hAOs were cultured with the test medium for 48 h, an Annexin V/PI assay was used to measure the cell apoptosis of hAOs; the hAOs were minimally washed with PBS, 5 μM Annexin V-FITC was added for 5 min, and then 5 μM of PI was added. The images were obtained using a confocal microscope (Leica, TCS SP8, Germany). All tests were performed in three replicates.

### 2.8 qPCR analysis

After depolymerized hAOs were cultured with the test medium for 48 h, total RNA was extracted using TRIzol reagent (Invitrogen, United States), and a NanoDrop One Spectrophotometer (NanoDrop Technologies, United States) was used to measure the RNA concentrations. Total mRNA was reverse-transcribed using ABScript III RT Master Mix for qPCR with gDNA remover (ABclonal, China). The qPCR reactions were performed on the ABI QuantStudio™ 7 Flex Real-Time PCR using System 2× Universal SYBR Green Fast qPCR Mix. All primer information is provided in Supplementary Table S1.

### 2.9 Statistical analysis

Data were analyzed by *t*-test and expressed as mean ± standard deviation (SD), and the level of significant difference was *p* < 0.05. All statistics were completed using GraphPad Prism 8.0.

## 3 Results

### 3.1 Generation and characterization of HBEC-derived hAOs

**FIGURE 2:**
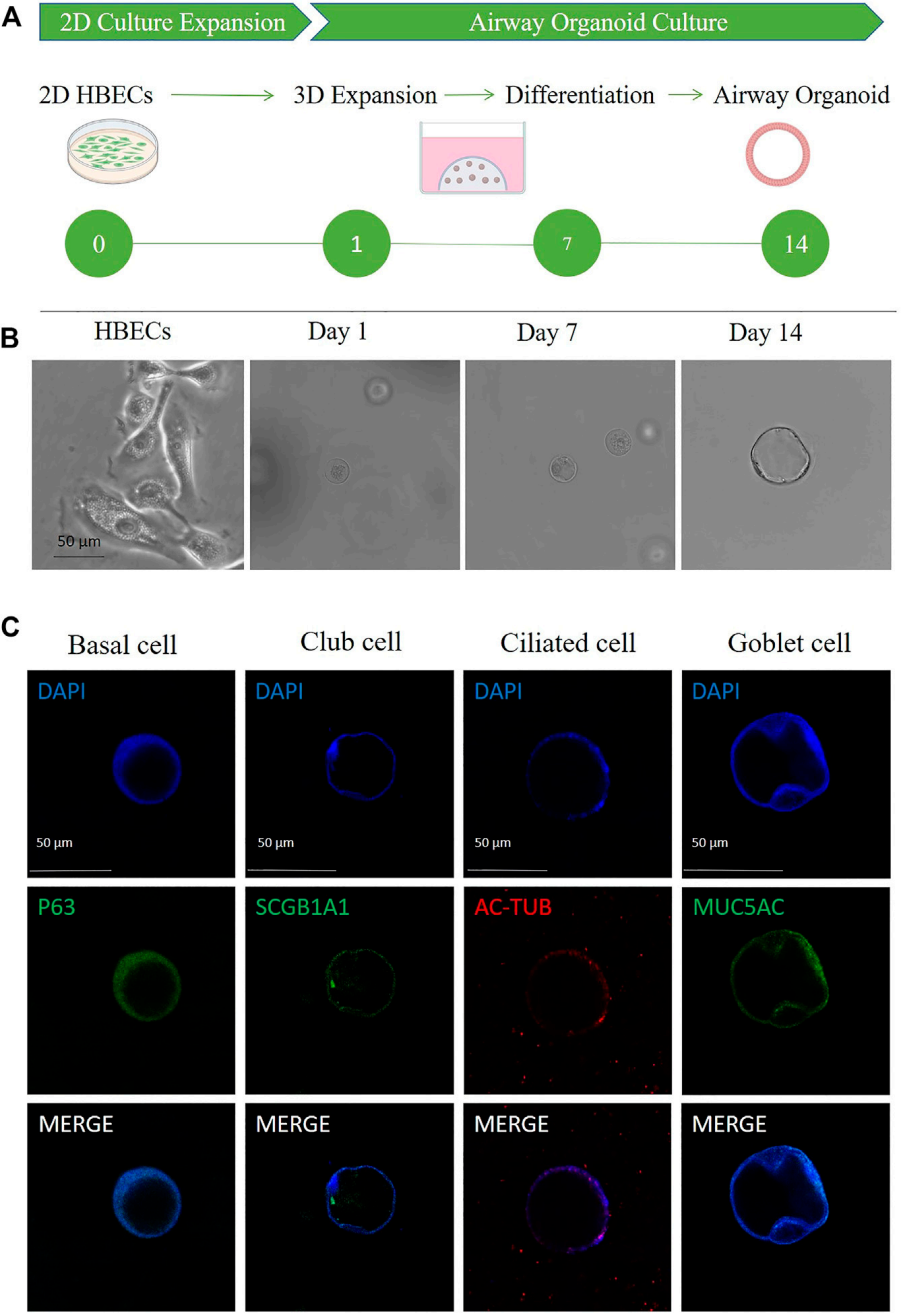
Characterization of hAOs. **(A,B)** Schematic of HBEC differentiation to hAOs. Scale bar, 50 μm. **(C)** Immunofluorescence of hAOs indicating specific markers for basal cells (p63), club cells (*SCGB1A1*), ciliated cells (Ac-tub), and goblet cells (MUC5AC). Nuclei were counterstained in blue. Scale bar, 50 μm.

A schematic representation and method summary of our study. HBECs were induced to differentiate and form hAOs as shown in Figure 2A. After embedded in Matrigel for 14 days, HBECs eventually formed a cavity-like sphere with a diameter of approximately 50 μm, which were composed of a polarized, pseudostratified airway epithelium (Figure 2B). hAOs were characterized by derived cells through examination of the expression of marker proteins to determine their expression, and basal (p63), club (*SCGB1A1*), ciliated (AC-tubulin), and goblet (MUC5AC) cell markers were positive (Figure 2C), which supports the fact that hAOs were successfully constructed.

### 3.2 Characterization of TWPs

**FIGURE 3:**
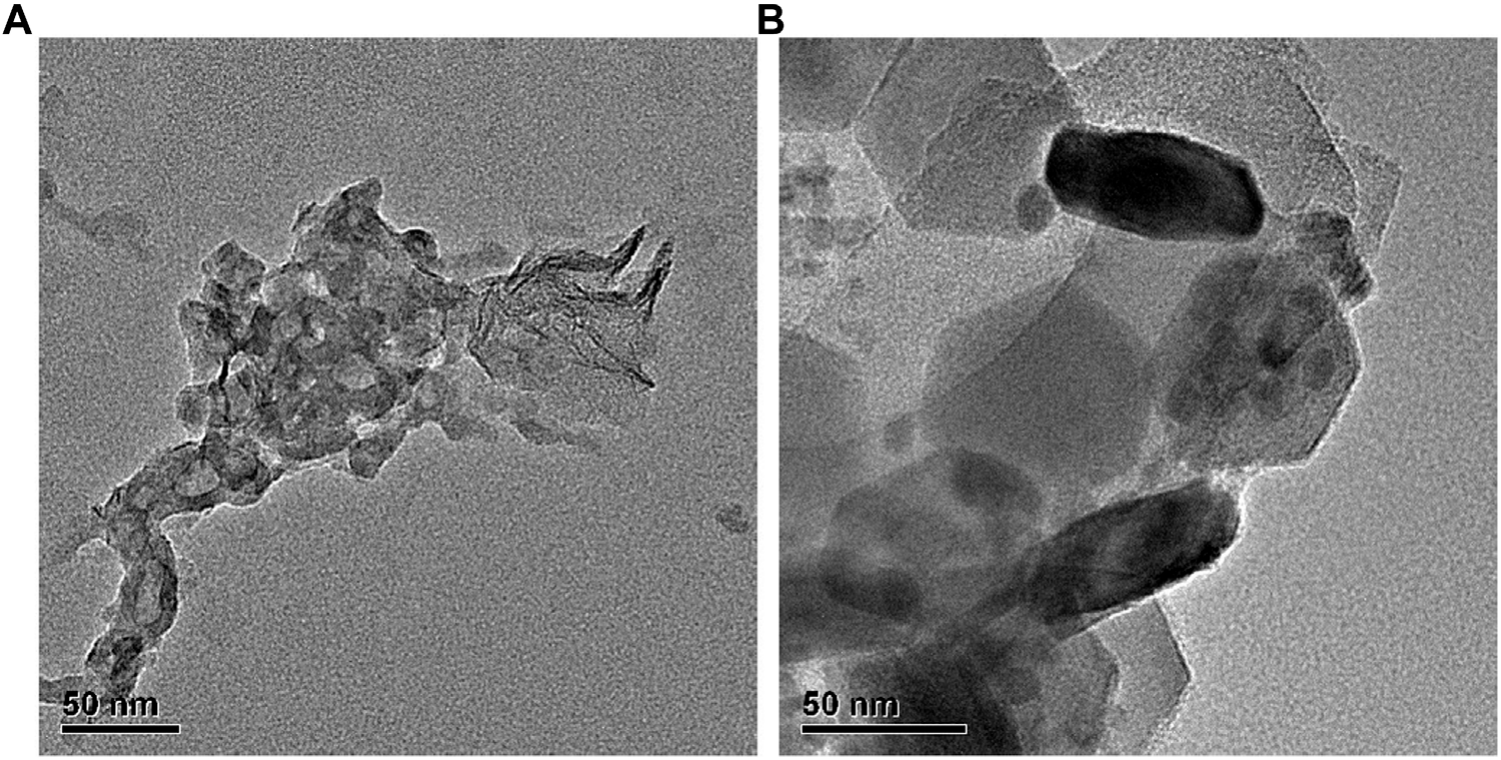
Scanning electron microscope image of TWPs. Scale bar, 50 nm.

Results from SEM showed that TWPs had very heterogenic forms, and they were elongated in shape and had sharp in edges. Moreover, the size of TWPs used in this study are approximately 100 nm (Figure 3).

### 3.3 The cytotoxicity of TWPs on HBECs

**FIGURE 4:**
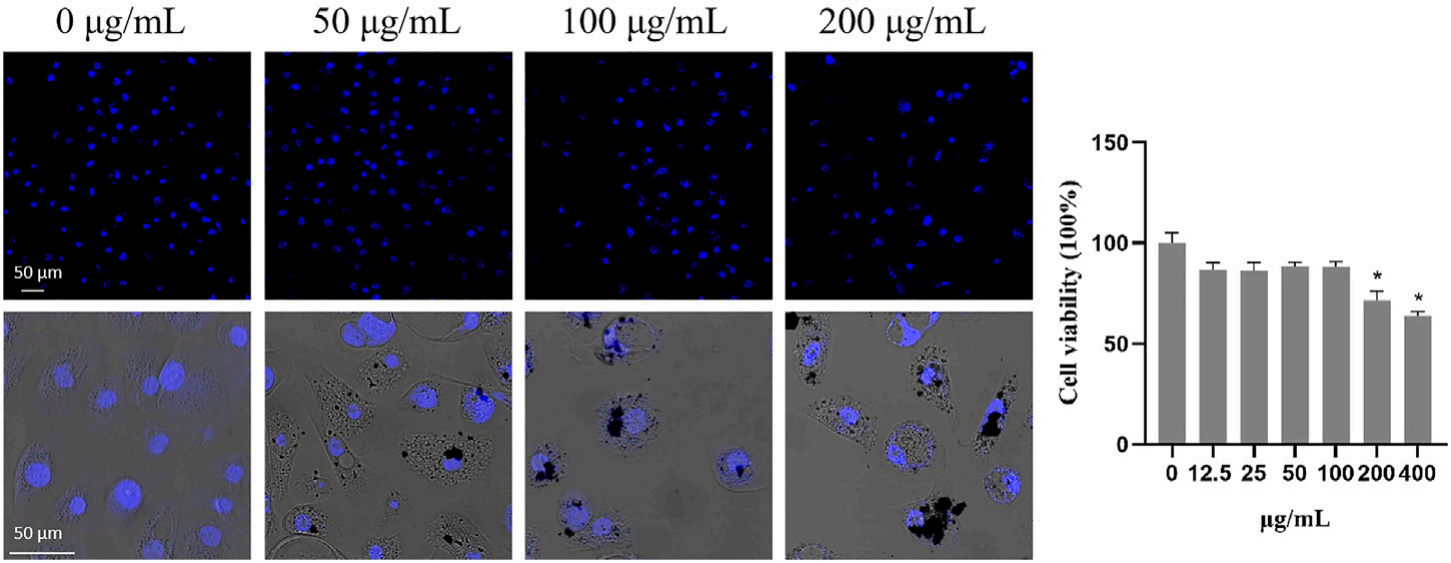
Cytotoxicity of TWPs on HBECs. Nuclei were stained by DAPI to determine the number of cells; scale bar, 50 μm. Data are presented as the cell viability normalized to the control. Values represent the mean ± SD of three independent experiments.

To investigate the cytotoxicity of TWPs on HBECs, HBECs were treated with the cells marked by DAPI and counted, and the results showed that the concentrations of TWPs higher than 200 μg/ml induced significant cytotoxicity on HBECs (Figure 4). Moreover, TWPs clustered around HBECs and even smaller TWPs were found in cells.

### 3.4 The effects of TWPs on the growth of hAOs

**FIGURE 5:**
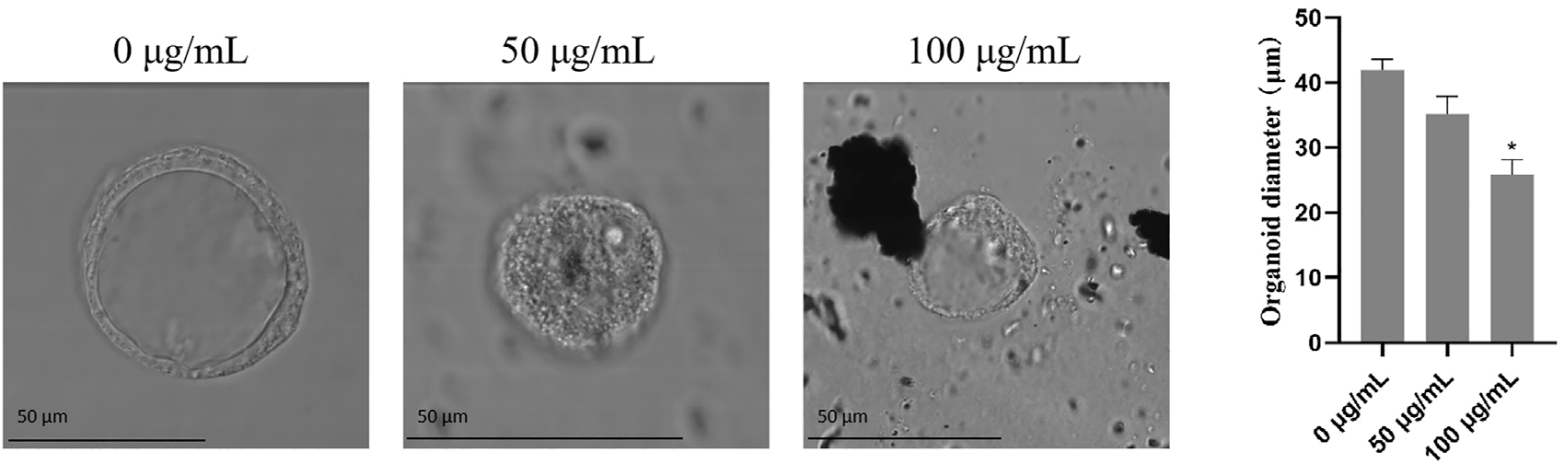
Effects of TWPs on the growth of hAOs. The morphology of hAOs was captured under bright field to determine the diameters of hAOs; scale bar, 50 μm.

To assess the effects of TWPs on the growth of hAOs, the size of hAOs was measured after exposure to TWPs for 24 h. Results suggest that although TWPs did not affect the spherical architecture of hAOs, the radial sizes of hAOs treated with 100 μg/ml TWPs were smaller than that of the control group (Figure 5).

### 3.5 The effects of TWPs on cell apoptosis of hAOs

**FIGURE 6:**
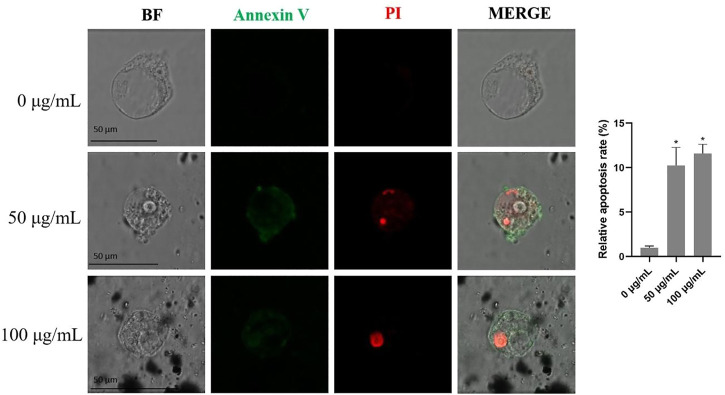
Cell apoptosis induced by TWPs in hAOs was detected by Annexin V-FITC/PI staining. Green fluorescence and red fluorescence represent early and late apoptotic cells, respectively. Scale bar, 50 μm.

The results from the Annexin V/PI apoptosis assays showed that TWPs exposure significantly elevated the proportions of early (green fluorescence) and late (red fluorescence) apoptotic cells in hAOs (Figure 6). Moreover, late apoptosis cells induced by TWPs were concentrated in the interior of the hAOs, and early apoptosis cells were mainly found in the periphery of hAOs (Figure 6).

### 3.6 The evaluation of TWPs on ROS generation and inflammation in hAOs

**FIGURE 7:**
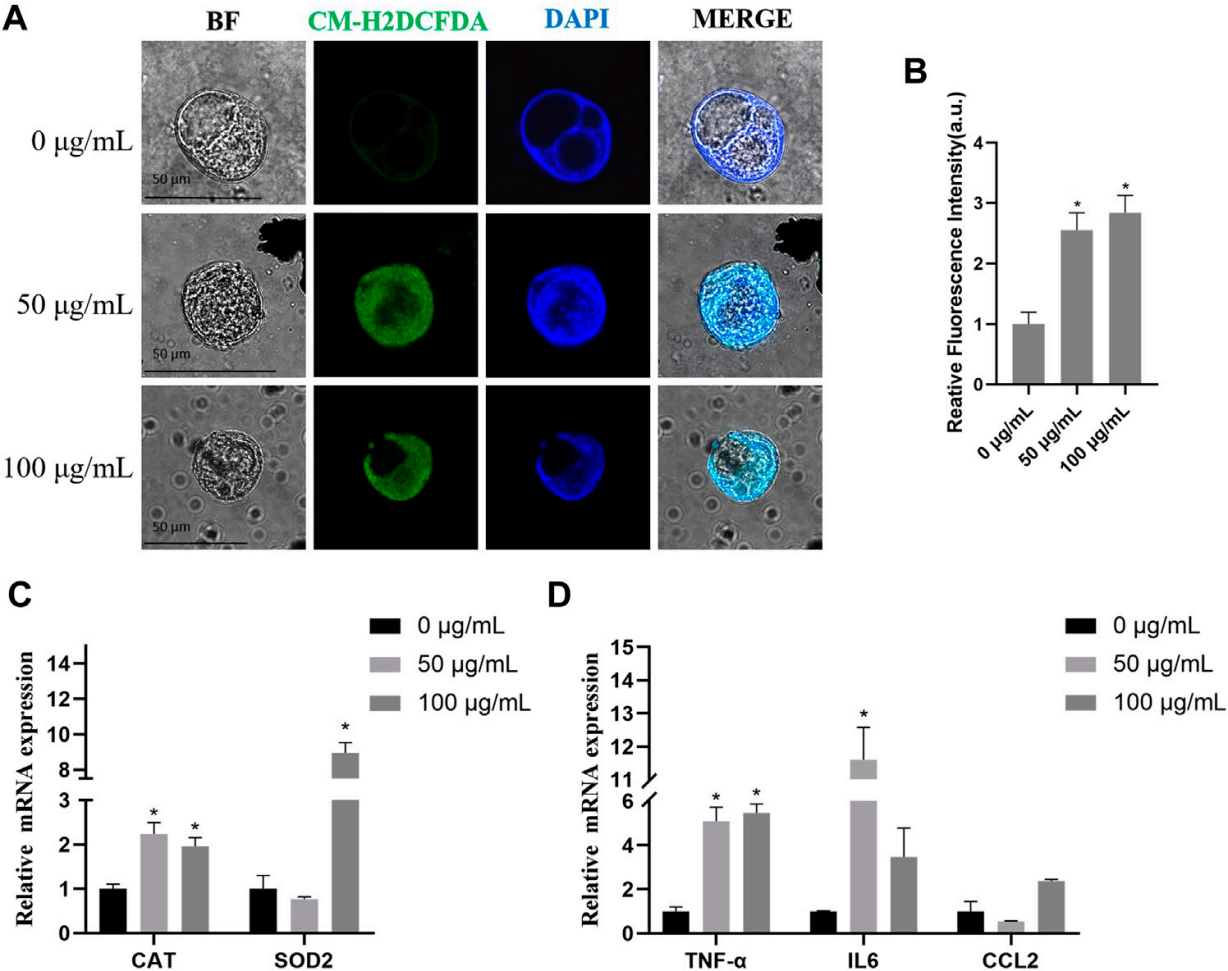
ROS generation and inflammation induced by TWPs in hAOs. **(A,B)** ROS production induced by TWPs in hAOs was detected by CM-H2DCFDA staining. Green fluorescence and blue fluorescence represent ROS and nuclei, respectively. Scale bar, 50 μm. ROS levels are presented as the relative fluorescence intensity normalized to the control. **(C,D)** qPCR analysis for oxidative stress-related genes and inflammation cytokine-related genes.

To study the effects of TWPs on oxidative stress, results from an ROS fluorescence probe showed that TWPs significantly induced ROS generation in a dose-dependent manner, and the ROS relative levels in 100 μg/ml TWPs were 2.83 fold higher than that of the control (Figures 7A, B). In qPCR, TWP exposure significantly increased oxidative stress-related gene expression including *CAT* and *SOD2*, and the increased inflammation of cytokine-related gene expression including TNFα, IL-6, and ccl2 was also observed in 100 μg/ml TWP exposure (Figures 7C, D).

### 3.7 The evaluation of TWPs on cell composition in hAOs

**FIGURE 8:**
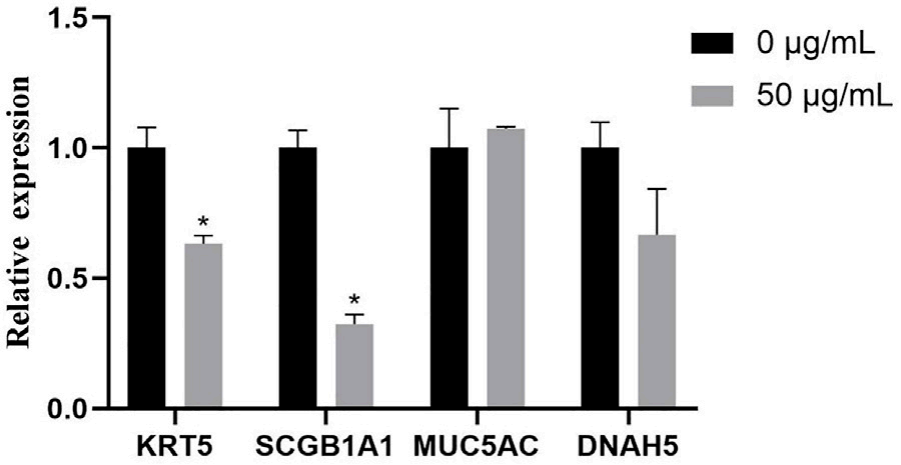
qPCR analysis for genes of hAO cell-specific markers for basal cells (*KRT5*), club cells (*SCGB1A1*), goblet cells (MUC5AC), and ciliated cells (DNAH5).

To evaluate the effects of TWPs on cell composition in hAOs, we used qPCR to determine the typical airway epithelial markers *KRT5* (basal cells), *SCGB1A1* (club cells), MUC5AC (goblet cells), and DNAH5 (ciliated cells), and the gene expression of *KRT5* and *SCGB1A1* was significantly decreased in the 50 μg/ml TWP exposure group (Figure 8).

## 4 Discussion

The upper airway epithelium, as the first line of defense of the human respiratory system, is constantly exposed to various air pollutants, and its injury can lead to the occurrence and aggravation of chronic respiratory diseases. Thus, using *in vitro* models adequately representing the human airway microenvironment to assess the toxic effects of air pollutants and identifying potentially harmful substances can help prevent these related diseases. In our study, we developed an *in vitro* 3D model derived from HBECs, which is similar to the airway structures, and then we used this model to evaluate the potential effects of emerging air pollutant TWPs on the human airway.

Growing evidence indicates that 3D human organoids provide unique opportunities for toxicology studies as organoids are similar to the corresponding *in vivo* organs in microenvironments as well as cellular interactions (M Li Y. et al., 2022; Xu et al., 2022). Liver organoids have been used to study the potential risks on liver steatosis and fibrosis of polystyrene microplastic particles (Cheng et al., 2022). The cardiac organoids were performed to screen effects on the beating rate of toxics such as Pb, mercury, and glyphosate (Forsythe et al., 2018). In brain and intestinal organoids, Cd displayed abnormal differentiation in neural and secretory cells, respectively (Yin et al., 2018; Xie et al., 2020). As for lung organoids, the 3D organoid model of alveolar type 2 epithelial cell-like cells (ATLs) induced by human pluripotent stem cells was developed to meet the urgent need for reliable and effective models to investigate the health effects of air pollutants on human lungs and it recapitulated key features of ATLs of secretory abilities (S Liu S. et al., 2021). Moreover, nano-SiO2 was taken up in a dose-dependent manner and interference with its surfactant secretion occurred in the toxicity evaluation. However, in addition to alveolar epithelial cells, the lungs consist of various airway epithelium cells. Our study reported 3D hAOs derived from HBECs for 14 days of differentiation, and they eventually formed cavity-like spheres including basal cells, functional multi-ciliated cells, goblet cells, and club cells. The hAOs closely mimic the human airway in terms of cellular diversity, spatial architecture, and function, which are available for air pollution toxicology screenings.

It was estimated that the ambient air of roads contained approximately 0.4–11 μg/m^3^ TWPs, which were found to contribute 11% of total traffic-derived PM_10_ mass (Gonet and Maher 2019). Additionally, it should not be overlooked that TWPs have become an emerging air pollutant. The size distribution of TWPs was reported to range from a few nanometers to several hundred micrometers (Wagner et al., 2018), and the size of TWPs measured by SEM in this study was approximately 100 nm, which indicated that TWPs carried a risk of inhalation. Moreover, the very heterogenic forms of TWPs were also observed and this suggests that TWPs had a great surface area per unit mass that delivered more soluble compounds to induce potential toxicity on the lungs.

Notably, non-tailpipe pollution, such as TWPs, is indicated as a possible connection to decreased lung function with −0.84% of FEV1 from baseline in non-asthmatic subjects (Krall et al., 2018). Because the airway epithelium, located between the host and the external environment, represents the first line of defense (Lambrecht and Hammad 2012), a breach in its integrity and an increase of mucus-producing goblet cells could induce FEV1 reduction (Athari 2019). Therefore, we speculate that TWPs could result in toxicity on the airway epithelium. To test this hypothesis, we first exposed HBECs that have not been induced to differentiation of TWPs. Our results revealed the TWP concentrations that are higher than 200 μg/ml had cytotoxicity on HBECs, and TWPs clustered around HBECs, which was similar to a previous study reported on lung epithelial cells coated with microplastic fibers from synthetic cloth and fabric (Winkler et al., 2022). However, 2D HBECs cultures (monolayers) lost tissue-specific functioning of the cells. To further determine the effects of TWPs on human airway microenvironments, 3D hAOs were treated with non-cytotoxic TWPs concentrations for 24 h, and the results showed that TWPs reduced the size of hAOs, which indicated that TWPs had inhibitory effects on hAO growth. In Annexin V/PI assays, TWP exposure leads to significant cell apoptosis in hAOs. The main mechanism of air pollution toxicity on the lungs was considered associated with their ability to induce oxidative stress, and air pollution disrupted a balance between ROS production and the anti-oxidant defenses (Crobeddu et al., 2017). Thus, a CM-DCFDA probe was performed to evaluate weather TWPs caused oxidative stress, and the results showed that TWPs induced significant ROS generation in a dose-dependent manner. When ROS levels were elevated, both the adaptive anti-oxidant response and inflammatory response could be triggered, and therefore, to further determine the effects of TWPs on anti-oxidant enzymes and inflammatory cytokines, the genes of *SOD2*, *CAT*, TNFα, IL-6, and CCL2 were studied, and their significant up-regulation of mRNA expression was observed, which suggests that TWPs exerted toxic effects on hAOs through oxidative stress and the inflammatory response.

The airway epithelium consisting of multiple cell types, including club, goblet, ciliated, and basal cells, is the protective barrier of the respiratory tract, and adequate and balanced cell composition is essential for an optimal airway microenvironment structure and functionality (Dean and Snelgrove 2018; Carrer et al., 2020). Balance disrupting was associated with the occurrence and progression of respiratory diseases. For example, long-term exposure to organic dust leads to increased goblet cell numbers and mucus over-production in the airways of workers, which resulted in a decrease in FEV1/FVC and promotion of the occurrence of chronic obstructive pulmonary disease (COPD) (Kim and Criner 2013). In our study, an alteration in the cellular balance was found, and most importantly, the gene expression of *KRT5* and *SCGB1A1* was significantly reduced. Basal cells marked by *KRT5* were the progenitor cells of ciliated cells and club cells, and TWPs induced decreased basal cells, which implicated that TWPs affected the differentiation of airway epithelial cells. *SCGB1A1* as a club cell secretory protein was a biologic marker of lung function alterations (Zhai et al., 2019; Johnson et al., 2021) and was demonstrated to be critical in mediating anti-inflammatory and antioxidant functions within the lungs. Decreased club cells increased airway sensitivity and resistance and have a positive association with a decline in FEV1 (Zhai et al., 2019). Interestingly, TWPs have been indicated with decreased lung function with −0.84% of FEV1 from baseline in non-asthmatic subjects (Krall et al., 2018), which implicated that it might be attributed to the loss of club cells induced by TWPs leading to airway damage, which is also worth further study. Meanwhile, the aforementioned results also emphasized the advantages of hAOs for a toxicity assessment and mechanistic studies. Compared with 2D cell models, hAOs were more suitable to comprehensively reflect the toxicity of air pollutants on human airway physiological microenvironments and cellular interactions. Moreover, various methodologies were provided for airborne pollutant toxicity screening using hAOs, and confocal microscopy was used to evaluate the overall toxicity on organoid morphology, such as the size of the volume, cell composition, and cell arrangement, and specific toxicological markers allowed for more detailed analysis of toxicological pathways such as oxidative stress and the inflammation response. However, to evaluate the toxicity of chemicals in a time-efficient manner, there is great potential to combine hAOs with high content screening to establish a rapid high-throughput screening method.

## 5 Conclusion

In this study, we reported the utilization of HBEC induction models for a 3D *in vitro* hAO model representing the human airway microenvironment to assess the toxicity of emerging air pollutant TWPs. We induced HBECs differentiation to generate hAOs, which consist of basal cells, ciliated cells, goblet cells, and club cells, and closely reflected the human airway cellular diversity and function. Our results indicate reduced size, significant cell apoptosis, oxidative stress, an inflammation response, and an imbalance of cell types in hAOs exposed to TWPs. Thus, we provide a 3D hAO model to address urgently needed human models for the assessment of air pollutants potentially affecting the airways, and hAOs were employed for the first time to evaluate the toxicity of TWPs on the lungs.

## Data Availability

The raw data supporting the conclusions of this article will be made available by the authors, without undue reservation.
