# Supplementary material for: Human airway organoids as 3D in vitro models for a toxicity assessment of emerging inhaled pollutants: Tire wear particles
Source: Front Bioeng Biotechnol. 2023 Jan 6;10:1105710. doi: 10.3389/fbioe.2022.1105710 (PMC9853070; doi:10.3389/fbioe.2022.1105710)
Supplement: Supplementary file 1 [file DataSheet1.doc]

**Supporting information**

Human airway organoids as 3D in vitro models for toxicity assessment of emerging inhaled pollutants: tire wear particles

Yingying Jiang^a,1^, Lin Lu ^a,1^, Chao Du ^a,1^, Yanting Li^a^, Wenting Cheng^a^, Huanhuan Bi^b^, Guo Li^b^, Min Zhuang^b^, Dunqiang Ren ^b^, Hongmei Wang ^b^,*, Xiaoya Ji ^a^,*

^a^ Department of Occupational and Environmental Health, School of Public Health, Qingdao University, Qingdao 266071, China

^b^ Department of Pulmonary and Critical Care Medicine, Affiliated Hospital of Medical College of Qingdao University, Qingdao 266021, China

* Corresponding author: [jxya@qdu.edu.cn](mailto:jxya@qdu.edu.cn) (Xiaoya Ji) or dor.whm@163.com (Hongmei Wang)

^1^ These authors contributed equally.

Table S1. Genes, forward (FWD) and reverse (REV) sequences of primers

| **Gene** | **Primer** | **Sequence** |
| --- | --- | --- |
| SOD2 | Forward | GCTCCCCGCGCTTTCTTA |
|  | Reverse | GCTGGTGCCGCACACT |
| CAT | Forward | TTGCCACAGGAAAGTACCCC |
|  | Reverse | TGAGGCCAAACCTTGGTGAG |
| TNF-a | Forward | GCCCATGTTGTAGCAAACCC |
|  | Reverse | TATCTCTCAGCTCCACGCCA |
| IL-6 | Forward | GCCCAGCTATGAACTCCTTCT |
|  | Reverse | GCAAGTCTCCTCATTGAATCCAG |
| CCL2 | Forward | CATGAAAGTCTCTGCCGCCC |
|  | Reverse | GGGCATTGATTGCATCTGGCTG |
| Krt5 | Forward | GCATCACCGTTCCTGGGTAA |
|  | Reverse | GACACACTTGACTGGCGAGA |
| Scgb1a11 | Forward | TCCTCCACCATGAAACTCGC |
|  | Reverse | AGGAGGGTTTCGATGACACG |
| DNAH5 | Forward | AGAGGCCATTCGCAAACGTA |
|  | Reverse | CCCGGAAAATGGGCAAACTG |
| Muc5ac | Forward | AGCCGGGAACCTACTACTCG |
|  | Reverse | AAGTGGTCATAGGCTTCGTGC |
| β-actin | Forward | CATCTACACAGTTTGATGCTGCT |
|  | Reverse | CATCTACACAGTTTGATGCTGCT |

Table S2. original data of qPCR

| gene | TWPs concentration (μg/ml) | CT | gene | TWPs concentration (μg/ml) | CT |
| --- | --- | --- | --- | --- | --- |
| β-ACTIN | 0 | 15.702 | β-ACTIN | 0 | 16.710 |
| β-ACTIN | 0 | 15.613 | β-ACTIN | 0 | 16.011 |
| β-ACTIN | 0 | 15.638 | β-ACTIN | 0 | 16.466 |
| β-ACTIN | 50 | 15.131 | β-ACTIN | 50 | 17.214 |
| β-ACTIN | 50 | 15.291 | β-ACTIN | 50 | 16.994 |
| β-ACTIN | 50 | 15.071 | β-ACTIN | 50 | 16.914 |
| β-ACTIN | 100 | 18.272 | β-ACTIN | 100 | 16.790 |
| β-ACTIN | 100 | 18.225 | β-ACTIN | 100 | 16.981 |
| β-ACTIN | 100 | 18.185 | β-ACTIN | 100 | 16.857 |
| KRT5 | 0 | 18.500 | DNAH | 0 | 33.763 |
| KRT5 | 0 | 18.673 | DNAH | 0 | 33.290 |
| KRT5 | 0 | 18.810 | DNAH | 0 | 34.013 |
| KRT5 | 50 | 18.806 | DNAH | 50 | 35.436 |
| KRT5 | 50 | 18.830 | DNAH | 50 | 34.247 |
| KRT5 | 50 | 18.851 | DNAH | 50 | 35.307 |
| MUC5AC | 0 | 33.602 | TNF-α | 0 | 30.593 |
| MUC5AC | 0 | 33.848 | TNF-α | 0 | 30.629 |
| MUC5AC | 0 | 34.308 | TNF-α | 0 | 30.038 |
| MUC5AC | 50 | 33.283 | TNF-α | 50 | 29.051 |
| MUC5AC | 50 | 33.424 | TNF-α | 50 | 28.287 |
| MUC5AC | 50 | 33.186 | TNF-α | 50 | 28.687 |
| SCGB1A1 | 0 | 28.608 | TNF-α | 100 | 28.192 |
| SCGB1A1 | 0 | 28.733 | TNF-α | 100 | 28.396 |
| SCGB1A1 | 0 | 28.868 | TNF-α | 100 | 28.600 |
| SCGB1A1 | 50 | 29.568 | CAT | 0 | 29.334 |
| SCGB1A1 | 50 | 30.022 | CAT | 0 | 29.094 |
| SCGB1A1 | 50 | 30.058 | CAT | 0 | 29.059 |
| CCL2 | 0 | 33.259 | CAT | 50 | 28.513 |
| CCL2 | 0 | 32.043 | CAT | 50 | 28.667 |
| CCL2 | 0 | 34.526 | CAT | 50 | 28.772 |
| CCL2 | 50 | 33.441 | CAT | 100 | 28.603 |
| CCL2 | 50 | 33.355 | CAT | 100 | 28.518 |
| CCL2 | 50 | 33.253 | CAT | 100 | 28.888 |
| CCL2 | 100 | 34.251 |  |  |  |
| CCL2 | 100 | 34.281 |  |  |  |
| CCL2 | 100 | 34.324 |  |  |  |
| IL6 | 0 | 31.772 |  |  |  |
| IL6 | 0 | 31.640 |  |  |  |
| IL6 | 0 | 31.754 |  |  |  |
| IL6 | 50 | 27.927 |  |  |  |
| IL6 | 50 | 27.678 |  |  |  |
| IL6 | 50 | 27.523 |  |  |  |
| IL6 | 100 | 33.345 |  |  |  |
| IL6 | 100 | 33.092 |  |  |  |
| IL6 | 100 | 31.650 |  |  |  |
| SOD2 | 0 | 32.966 |  |  |  |
| SOD2 | 0 | 32.275 |  |  |  |
| SOD2 | 0 | 33.959 |  |  |  |
| SOD2 | 50 | 32.945 |  |  |  |
| SOD2 | 50 | 32.934 |  |  |  |
| SOD2 | 50 | 32.562 |  |  |  |
| SOD2 | 100 | 31.882 |  |  |  |
| SOD2 | 100 | 32.500 |  |  |  |
| SOD2 | 100 | 32.132 |  |  |  |


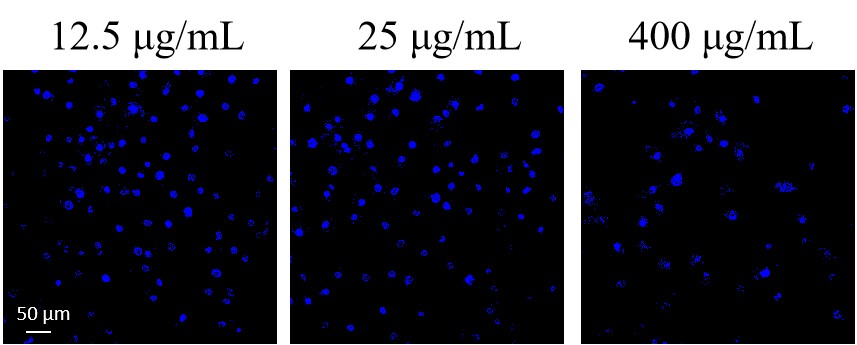


Figure S1. The cytotoxicity of 12.5, 25, 400 ug/ml TWPs on HBECs. Nuclei were stained by DAPI for determining the number of cells; scale bar: 50 μm
